# Regulation of reverse cholesterol transport - a comprehensive appraisal of available animal studies

**DOI:** 10.1186/1743-7075-9-25

**Published:** 2012-03-29

**Authors:** Wijtske Annema, Uwe JF Tietge

**Affiliations:** 1 Department of Pediatrics, Center for Liver, Digestive and Metabolic Diseases, University of Groningen, University Medical Center Groningen, Groningen, The Netherlands; 2 Top Institute Food and Nutrition, Wageningen, The Netherlands

**Keywords:** Atherosclerosis, Bile, Cholesterol, Efflux, Feces, High density lipoproteins, Intestine, Liver, Macrophages, reverse cholesterol transport

## Abstract

Plasma levels of high density lipoprotein (HDL) cholesterol are strongly inversely correlated to the risk of atherosclerotic cardiovascular disease. A major recognized functional property of HDL particles is to elicit cholesterol efflux and consequently mediate reverse cholesterol transport (RCT). The recent introduction of a surrogate method aiming at determining specifically RCT from the macrophage compartment has facilitated research on the different components and pathways relevant for RCT. The current review provides a comprehensive overview of studies carried out on macrophage-specific RCT including a quick reference guide of available data. Knowledge and insights gained on the regulation of the RCT pathway are summarized. A discussion of methodological issues as well as of the respective relevance of specific pathways for RCT is also included.

## What is the relevance of reverse cholesterol transport?

Large population studies conclusively demonstrated that plasma levels of high density lipoprotein cholesterol (HDL-C) as well as its major apolipoprotein constituent apolipoprotein A-I (apoA-I) are inversely associated with the risk of atherosclerotic cardiovascular disease [1-4]. However, within these study populations there is still a substantial number of patients that experience complications of cardiovascular disease despite considerably high HDL-C plasma levels [1,2,4], and vice versa there are individuals with low plasma HDL-C levels that do not develop clinically significant atherosclerosis [1,2,4]. Such observations lead to the investigation how HDL particles confer protection against atherosclerosis. One of the earliest recognized functions of HDL is that it promotes cholesterol efflux from macrophage foam cells, which constitute the hallmark cell type of atherosclerotic lesions [5,6]. Upon entrance into the vessel wall monocytes become macrophages and take up vast amounts of modified pro-atherogenic apoB-containing lipoproteins that are accumulating within the vascular wall as an early event in the process of atherogenesis [7,8]. Uptake of cholesterol immobilizes macrophages within the vessel wall resulting in a sustained inflammatory response [8,9]. Importantly, cholesterol efflux from foam cells can revert this phenotype leading to macrophage egress from lesions and a subsequent reduction in lesion burden [10]. HDL-mediated cholesterol efflux therefore constitutes a key step not only for preventing lesion progression but also for clinical efforts to induce regression of preexisting atherosclerotic plaques. Subsequently, the cholesterol effluxed from foam cells towards HDL should ideally be irreversibly eliminated from the body to prevent re-uptake into the vessel wall. This goal is achieved by a complex multistep process that has been coined reverse cholesterol transport (RCT) [5,10,11].

## What is reverse cholesterol transport?

**Figure 1:**
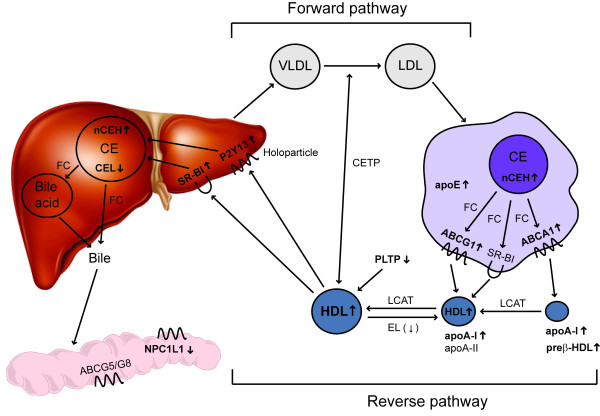


Reverse cholesterol transport is a term that comprises all the different steps in cholesterol metabolism between cholesterol efflux from macrophage foam cells and the final excretion of cholesterol into the feces either as neutral sterols or after metabolic conversion into bile acids (see Figure 1) [5,10,11].

The liver plays a central role in cholesterol metabolism. Cholesterol either derived from the diet or from synthesis within the liver or intestine is secreted by hepatocytes in the form of apoB-containing lipoproteins in a forward pathway to supply cholesterol to peripheral cells [10]. When chemically modified, these lipoproteins are taken up by macrophages resulting in foam cell formation [8,9]. From macrophages cholesterol can be effluxed as free cholesterol either via ATP binding cassette transporter A1 (ABCA1) with poorly lipidated apoA-I as acceptor or via ABCG1 with more mature spherical HDL particles serving as acceptor [6,11]. Additional efflux capacity might be provided by scavenger receptor class B type 1 (SR-BI) or by so-called aqueous diffusion [6,11]. Within HDL, cholesterol is esterified by lecithin-cholesterol acyltransferase (LCAT) thereby clearing space on the HDL surface for the uptake of additional free cholesterol [12]. Via the plasma compartment the effluxed cholesterol is transported in a reverse pathway back to the liver. Following receptor-mediated uptake of HDL cholesterol into hepatocytes either selectively via SR-BI or as a holoparticle via an as yet not fully characterized pathway [5], HDL-derived cholesterol is then de-esterified and secreted into the bile. This can occur either as free cholesterol or as bile acids. Notably, not in mice and rats but in humans, rabbits, hamsters and a number of other species expression of cholesteryl ester transfer protein (CETP) provides a shunt between the forward and the reverse cholesterol transport pathways [13]. This way also hepatic receptors for apoB-containing lipoproteins might participate in RCT. However, the differential relevance of the apoB-containing lipoprotein pathway versus the HDL pathway for RCT in humans is thus far unclear. Finally, within the intestinal lumen altered absorption rates of cholesterol can then further impact on the amount of foam cell-derived cholesterol that is finally excreted from the body [10].

## How can reverse cholesterol transport be quantified?

Initial attempts to quantify RCT used mass measurements of centripetal cholesterol flow from extrahepatic organs to the liver [14-16]. In addition, isotope techniques were employed to assess the dilution of an administered tracer over time by tissue-derived cholesterol [17]. However, all of these methods are not able to specifically trace cholesterol derived from macrophage foam cells, a small but highly relevant pool for atherosclerotic cardiovascular disease (CVD).

**Figure 2:**
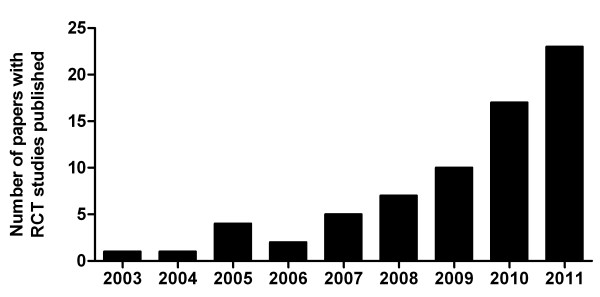


In 2003 the RCT field took up speed after Rader and colleagues introduced a novel *in vivo *method to specifically trace the movement of cholesterol from macrophages to plasma, liver, and feces (Figure 2) [18]. Briefly, macrophages are loaded *in vitro *with modified low density lipoproteins (LDL) and ^3^H-cholesterol to generate macrophage foam cells. After an equilibration period, the macrophages are injected intraperitoneally into recipient mice. Plasma samples are taken on several time points, and feces are collected continuously during the duration of the experiment. Although this only represents a fraction of the total injected dose within the time frame of a given experiment, completed RCT is defined as the amount of ^3^H-tracer originating from macrophages that is recovered within feces. Of note, a potentially new experimental approach with macrophage loading *in vivo *using ^3^H-cholesteryl oleate-labeled oxidized LDL was recently communicated at scientific conferences [19], which will, however, not be further discussed in the present review.

## Which macrophages should be used in *in vivo *reverse cholesterol transport studies?

Since its first description in 2003, the technique to measure macrophage-to-feces RCT has become a widely accepted and frequently applied method. However, differences exist in the type of macrophages injected into the recipient animals, either cell lines or primary macrophages are used.

Most experiments published so far using cell lines employed murine J774 macrophages. The second most popular cell line utilized to investigate RCT is the murine macrophage-like RAW 264.7 cell line. In addition, RCT studies have also been performed with mouse P388D1 macrophages. A major shortcoming of J774 macrophages is that ABCA1 is almost not expressed in these cells, and only after stimulation with cyclic AMP J774 cells express detectable levels of this major efflux transporter [20]. Moreover, no endogenous apoE production has been found in the J774 as well as in the RAW cells [21-23], whereas macrophage-apoE has been shown to significantly impact *in vivo *RCT [24]. Importantly, the responsiveness of RAW cells to liver X receptor (LXR) activation, one of the strongest stimuli of cholesterol efflux, is severely impaired due to a significant reduction in the expression of LXRβ and almost absent expression of LXRα [25,26]. In addition, the majority of the reported macrophage-to-feces RCT experiments are conducted in mice on a C57BL/6 background, while J774 and RAW 264.7 cells originate from BALB/c mice, and P388D1 cells were originally derived from DBA/2 mice. Therefore, an impact of immunological incompatibilities cannot formally be excluded. Finally, an important disadvantage associated with macrophage cell lines is that changes in cellular characteristics may occur over time in culture. Nonetheless, the passage number of the cells is often not mentioned in the description of the RCT method.

Besides macrophage cell lines, RCT assays are also carried out with primary macrophages obtained from either the bone marrow or the peritoneal cavity. Primary macrophage cells have characteristics that more closely conform to macrophages *in vivo*, and thereby provide in our view more physiologically relevant results. Moreover, isolation of primary macrophage cells from genetically modified (knockout or transgenic) mice offers the opportunity to investigate the impact of specific macrophage-derived factors on *in vivo *RCT. However, it should be considered that significant differences in the degree of lipid loading may exist depending on the macrophage genotype, and that this might conceivably translate into alterations in macrophage RCT *in vivo *due to effects unrelated to the RCT pathway.

## What are limitations in the interpretation of results from macrophage reverse cholesterol transport assays?

Depending on the type of macrophages used (please see above), tracer recovery within feces varies between less than 1% (cell lines) and up to 10% (primary macrophages) within the time frame of a RCT experiment. If these quantitative differences between experimental set-ups are qualitatively relevant resulting into different conclusions drawn from a specific intervention in the RCT pathway is currently not clear, since no comparative studies have been carried out. The points whether the tracer is appearing in plasma first in the unesterified form, which is to be expected, as well as the rate and speed of subsequent esterification have also not been formally addressed, yet. Furthermore, the current RCT methodology was designed to measure the unidirectional transport of cholesterol from macrophages to feces and neither allows assessment of tracer unloading nor determination of cholesterol influx. Thereby, a situation could be envisioned where increased fecal excretion of the tracer in a RCT study is not associated with increased unloading of administered macrophages, e.g. by factors impacting on intestinal cholesterol absorption (please see below). Therefore, methodological improvements are awaited in order to address these questions. Recently, Smith *et al. *put forward a modified *in vivo *RCT assay to enable quantification of the bidirectional flux of macrophage cholesterol [27]. For this procedure the cholesterol-laden macrophages are immobilized in Matrigel and then introduced subcutaneously in experimental animals. Several days after implantation, the Matrigel plugs are removed, and the cells are recovered for analysis of changes in cellular cholesterol and DNA content. Simultaneously, macrophage-specific RCT can be assessed. In addition, another approach to measure *in vivo *changes in cholesterol mass in macrophage foam cells concurrently with macrophage RCT was published [28]. This protocol involves entrapment of lipid and ^3^H-cholesterol-laden macrophages into semipermeable hollow fibers. Subsequently, the fibers containing macrophage foam cells are implanted in the peritoneal cavity of recipient mice. After 24 hours, the fibers are removed and the cells are assayed for protein and cholesterol mass content [28].

However, all macrophage RCT methods employed so far use cells administered at locations outside the vessel wall. All such experiments are therefore based on the main assumption that any given location is equal to or at least a close surrogate of the situation within the vascular wall. However, in atherosclerotic lesions additional factors conceivably have an impact not reflected in the current RCT assay methodology such as accessibility by the HDL particle, hypoxia or pH changes just to name a few.

## Which factors influencing single steps in the reverse cholesterol transport pathway have been identified so far?

From the different steps that are important in the RCT pathway, overall RCT might be differentially affected on different levels (see also Table 1 for a summary). To date several factors affecting only one single step of the pathway but impacting on total RCT have been identified. These can be divided into effects at the level of (i) the macrophage, (ii) the transport of cholesterol through the plasma compartment, (iii) the uptake by the liver, (iv) the excretion into the intestine, and (v) the excretion from the body.

**Table 1:** Quick reference guide: Overview of available reverse cholesterol transport studies

Factor investigated	Intervention	Animal model	Type of macrophages used	Effect on RCT	Ref
*Macrophage*					
ApoE	ApoE knockout macrophages	Wild-type mice	Peritoneal	↓	[24]
CE hydrolysis	Human nCEH transgenic macrophages	LDLr knockout mice	Peritoneal	↑	[29]
ABCA1	ABCA1 knockout macrophages	Wild-type mice	Bone marrow	↓	[30,31]
ABCG1	ABCG1 overexpression in macrophages	Wild-type mice	J774	↑	[31]
	ABCG1 knockdown in macrophages	Wild-type mice	J774	↓	[31]
	ABCG1 knockout macrophages	Wild-type mice	Bone marrow	↓	[31]
ABCA1/ABCG1	ABCA1/ABCG1 double knockdown in macrophages	Wild-type mice	J774	↓	[31]
	ABCA1/ABCG1 double knockout macrophages	Wild-type mice	Bone marrow	↓	[32]
SR-BI	SR-BI knockout macrophages	Wild-type mice	Bone marrow	=	[31]
	SR-BI knockout macrophages	Wild-type mice	Bone marrow	=	[33]
ABCA1/SR-BI	ABCA1/SR-BI double knockout macrophages	Wild-type mice	Bone marrow	↓	[33]
PLTP	PLTP knockout macrophages	Wild-type mice	Peritoneal	=	[34]
CETP	CETP overexpression in macrophages	Wild-type mice	RAW 267.4	=	[35]
	CETP overexpression in macrophages	Wild-type mice	Peritoneal	=	[36]
15(*S*)-lipoxygenase-1	Human 15(*S*)-lipoxygenase-1 overexpression in macrophages	Wild-type mice	RAW 267.4	↑	[37]
Myeloid differentiation primary response protein 88	MyD88 knockout macrophages	Wild-type	Peritoneal	↓	[38]
*Transport through the plasma compartment*					
ApoA-I	Adenoviral overexpression human apoA-I	Wild-type mice	J774	↑	[18]
	ApoA-I knockout	LDLr/apobec double knockout mice	J774	↓	[39]
	Adenoviral overexpression mouse or human apoA-I (wild-type or apoA-I Milano)	ApoA-I knockout mice	J774	↑	[40,41]
	Ro 11-1464	Human apoA-I transgenic mice	J774	↑	[42]
ApoA-I tertiary structural domain	AAV overexpression domain-swap variants of human and mouse apoA-I	ApoA-I knockout mice	J774	↑	[40]
ApoA-I Milano versus wild-type apoA-I	AAV overexpression	ApoA-I knockout mice	J774	=	[41]
ApoA-I mimetic peptides	D-4 F	ApoE knockout mice	J774	↑	[43]
	5A	Wild-type mice	RAW 264.7	↑	[17]
	ATI-5261	ApoE knockout mice	J774	↑	[44]
HDL particle formation	ABCA1 knockout	ABCA1 knockout mice	Endogenous	↓	[45]
	ABCA1 knockout	ABCA1 knockout mice	Peritoneal	↓	[33]
	Probucol	Wild-type mice	J774	=	[46]
	Probucol	SR-BI knockout mice	J774	↑	[46]
LCAT	AAV overexpression human LCAT	Human apoA-I transgenic mice	J774	↓	[47]
	AAV overexpression human LCAT	Human apoA-I transgenic mice overexpressing SR-BI	J774	=	[47]
	AAV overexpression human LCAT	Human apoA-I transgenic mice overexpressing human CETP	J774	=	[47]
	LCAT knockout	LCAT knockout mice	J774	↓	[47]
Hepatic lipase	HL knockout	HL knockout mice	J774	=	[48]
Endothelial lipase	EL knockout	EL knockout mice	J774	=	[48]
	Inhibition hepatic proprotein convertases	Wild-type mice	J774	↓	[49]
Hepatic lipase/endothelial lipase	HL/EL double knockout	HL/EL double knockout mice	J774	=	[48]
PLTP	PLTP overexpression	Human PLTP transgenic mice	Peritoneal	↓	[34]
CETP	Adenoviral CETP overexpression	Wild-type mice	RAW 264.7	↑	[35]
	AAV overexpression human CETP	Apobec knockout mice	J774	↑	[50]
	AAV overexpression human CETP	LDLr/apobec double knockout mice	J774	=	[50]
	AAV overexpression human CETP	SR-BI knockout mice	J774	↑	[50]
	CETP overexpression	Cynomolgus monkey CETP transgenic mice	P388D1 or peritoneal	=	[36]
	CETP overexpression	Human CETP transgenic mice	Bone marrow	=	[51]
CETP inhibition	Torcetrapib	Hamsters	J774	↑	[35]
	Torcetrapib	Hamsters	Peritoneal	↑	[52]
	Torcetrapib	Human CETP/human apoB100 transgenic mice	J774	↑	[53]
	Anacetrapib	Hamsters	Peritoneal	=	[52]
	Anacetrapib	Hamsters	J774	↑	[54]
	Dalcetrapib	Hamsters	Peritoneal	↑	[52]
ApoA-II	Overexpression human apoA-II	Human apoA-II transgenic mice	P388D1	↑ or =	[55]
ApoF	AAV overexpression mouse apoF	Wild-type mice	J774	=	[56]
*Uptake by the liver*					
Selective uptake	Adenoviral overexpression SR-BI	Wild-type mice	J774	↑	[57]
	Adenoviral overexpression SR-BI	Human apoA-I transgenic mice	J774	↑	[57]
	SR-BI knockout	SR-BI knockout mice	J774	↓	[57]
	SR-BI knockout	SR-BI knockout mice	Peritoneal	↓	[33]
	SR-BI knockout	SR-BI knockout mice	Bone marrow	↓	[51]
	Transgenic CETP overexpression	SR-BI knockout mice	Bone marrow	=	[51]
	Liver-specific SR-BI knockout	SR-BI conditional knockout mice	Bone marrow	↓	[51]
	Transgenic CETP overexpression	SR-BI conditional knockout mice	Bone marrow	=	[51]
Holoparticle uptake	P2Y_13 _knockout	P2Y_13 _knockout mice	Peritoneal	↓	[58]
Type 1 diabetes mellitus	Alloxan	Wild-type mice	Peritoneal	↓	[59]
	Streptozotocin	Wild-type mice	J774	↓	[60]
	Streptozotocin	Hp2-2 mice	J774	↓	[60]
*Excretion into the intestine*					
CE hydrolysis liver	Adenoviral overexpression human nCEH	Wild-type mice	J774	↑	[61]
	Carboxyl ester lipase knockout	Carboxyl ester lipase knockout mice	J774	↑	[62]
ABCG5/ABCG8	ABCG5/ABCG8 double knockout	ABCG5/ABCG8 double knockout mice	P388D1	=	[63]
Biliary sterol secretion	Bile duct ligation	Wild-type mice	Peritoneal	↓	[64]
	Surgical biliary diversion	Wild-type mice	J774	=	[65]
	Transgenic overexpression NPC1L1 in the liver	Liver-specific human NPC1L1 transgenic mice	J774	=	[65]
MDR2	MDR2 knockout	MDR2 knockout mice	Peritoneal	↓	[64]
*Excretion from the body*					
NPC1L1 intestine	Ezetimibe	Wild-type mice	J774	↑	[66]
	Ezetimibe	Wild-type mice	RAW 264.7	↑	[67]
Cholesterol absorption intestine	Congenic 14DKK interval	14DKK congenic mice	RAW 264.7	↑	[67]
	Congenic 14DKK interval	14DKK apoE knockout congenic mice	Bone marrow	↑	[68]
*Inflammation*					
Acute inflammatory response	LPS	Wild-type mice	Peritoneal	↓	[69]
	LPS	Wild-type mice	J774	↓	[70]
	Zymosan	Wild-type mice	RAW 264.7	↓	[71]
Human group IIA secretory phospholipase A_2_	Overexpression human group IIA secretory phospholipase A_2_	Human group IIA secretory phospholipase A_2 _transgenic mice	Peritoneal	=	[69]
Serum amyloid A	Adenoviral overexpression human serum amyloid A	Wild-type mice	Peritoneal	=	[69]
	Adenoviral overexpression mouse serum amyloid A	Wild-type mice	Peritoneal	↓	[69]
Myeloperoxidase	Human myeloperoxidase	Wild-type mice	Peritoneal	↓	[69]
Mast cell activation	Mast cell degranulating compound 48/80	Wild-type mice treated with human apoA-I	J774	↓	[72]
*Drugs*					
LXR agonist	GW3965	Wild-type mice	J774	↑	[73,74]
	GW3965	LDLr/apobec double knockout mice	J774	↑	[73]
	GW3965	ApoB/CETP double transgenic mice	J774	↑	[73]
	T0901317	Wild-type BALB/c mice	J774	↑	[75]
	GW3965	Hamsters	J774	↑	[76]
	T0901317	Wild-type mice	P388D1	↑	[63]
	T0901317	ABCG5/ABCG8 double knockout mice	P388D1	=	[63]
	T0901317	Wild-type FVB mice	Peritoneal	↑	[64]
	T0901317	MDR2 knockout mice	Peritoneal	=	[64]
	GW3965	LXR double knockout mice	J774	=	[74]
	GW3965	Wild-type mice	Bone marrow (wild-type and LXR double knockout)	=	[74]
Intestine-specific LXR activation	Constitutively activated LXRα in the intestine	iVP16LXRα transgenic mice	J774	↑	[77]
Liver-specific LXR activation	Adenoviral overexpression constitutively active LXRα	Wild-type FVB mice	J774	=	[77]
Intestine-specific LXR agonist	GW6340	Wild-type mice	J774	↑	[74]
PPARα agonist	GW7647	Wild-type mice	J774	↑	[78]
	GW7647	Human apoA-I transgenic mice	J774	↑	[78]
	GW7647	LDLr/apobec double knockout mice	J774	↑	[78]
	GW7647	Human apoA-I transgenic mice	Bone marrow (PPARα knockout)	=	[78]
	GW7647	Human apoA-I transgenic mice	Bone marrow (LXR double knockout)	=	[78]
	Fenofibrate	Human apoA-I transgenic mice	P388D1	↑	[79]
	Gemfibrozil	Human apoA-I transgenic mice	P388D1	=	[79]
PPARδ agonist	GW0742	Wild-type mice	J774	↑	[66]
PPARγ agonist	GW7845	Wild-type mice	J774	↓	[80]
FXR agonist	GW4064	Wild-type mice	J774	↑	[81]
	GW4064	SR-BI knockout mice	J774	↑	[81]
Liver-specific FXR activation	Adenoviral overexpression constitutively active FXR	Wild-type mice	J774	↑	[81]
miR-33 antagonism	Anti-miR33 oligonucleotide	LDLr knockout mice	Bone marrow	↑	[82]
Liver-selective thyromimetic	T-0681	Wild-type mice	J774	↑	[83]
	T-0681	CETP transgenic mice	J774	=	[83]
Sylfonylurea agent	Glibenclamide	Wild-type mice	RAW 264.7	=	[84]
	Glimepiride	Wild-type mice	RAW 264.7	=	[84]
Anti-HIV drug	Nevirapine	Human apoA-I transgenic mice	J774	=	[85]
	Efavirenz	Human apoA-I transgenic mice	J774	↑	[85]
Phosphodiesterase 3 inhibition	Cilostazol	Wild-type mice	RAW 264.7	↑	[86]
Proteasomal inhibition	Bortezomib	Wild-type mice	RAW 264.7	↑	[87]
Propolis extract	Ethanolic extract of propolis	Wild-type mice	RAW 264.7	↑	[88]
*Dietary modifications*					
Dietary cholesterol	Cholesterol-enriched diet	Hamsters	Peritoneal	↓	[89]
Dietary cholesterol and fat	Saturated fatty acid- and cholesterol-enriched diet	Wild-type mice	P388D1	↑	[90]
	Saturated fatty acid-enriched diet	Wild-type mice	P388D1	=	[90]
Coffee	Coffee	Wild-type mice	RAW 264.7	=	[91]
	Ferulic acid	Wild-type mice	RAW 264.7	↑	[91]
Fish oil	Fish oil	Wild-type mice	J774	↑	[92]
*Exercise*					
Exercise	Voluntary wheel running	Wild-type mice	Peritoneal	=	[93]
	Monitored wheel running	Human CETP transgenic mice	J774	↑	[94]

Wild-type; if not otherwise stated this term refers to the use of C57BL/6 mice

### What proteins influencing macrophage cholesterol metabolism are relevant for reverse cholesterol transport?

The first important step in the RCT pathway comprises the removal of excessive cholesterol from macrophage foam cells. The rate of cholesterol movement from macrophages to plasma is determined in the first place by the transport capacity of the macrophage. Cholesterol can be effluxed from the macrophage only in the unesterified or free form, but not as cholesteryl ester (CE). CEs stored in cytoplasmic lipid droplets of macrophages are hydrolyzed by a neutral cholesteryl ester hydrolase (nCEH) [95], and increased CE hydrolysis in lipid-laden macrophages by overexpression of human nCEH resulted in enhanced efflux of cholesterol [29]. However, such a manipulation is also likely to impact the cholesterol loading of the macrophages used in the RCT experiment. Nevertheless, *in vivo *the movement of radiolabeled cholesterol from macrophages into feces was significantly higher from macrophages expressing human nCEH [29], suggesting that efficient hydrolysis of intracellular CEs in macrophages is critical for the first step in RCT.

Free cholesterol can leave the macrophage by different pathways, which either might be transporter-independent (aqueous diffusion) or dependent on cholesterol transporters (SR-BI, ABCA1, and ABCG1). Ablation of ABCA1 specifically in macrophages decreased the flux of labeled cholesterol from macrophage foam cells into the serum as well as the feces [30,31]. Furthermore, RCT from macrophages was higher in wild-type mice after injection with macrophages overexpressing ABCG1 and significantly mitigated when using macrophages with reduced or no ABCG1 expression [31]. Importantly, macrophage ABCA1 and ABCG1 appear to function in concert in the *in vivo *RCT process, as knockdown of both ABCA1 and ABCG1 in macrophages suppressed macrophage-to-feces RCT more than either ABCA1 or ABCG1 deletion alone [31,32]. The general view is that apoA-I is lipidated by ABCA1 activity to generate nascent HDL particles, that then act as an acceptor for ABCG1-mediated cholesterol transport from macrophages. In addition to ABCA1- and ABCG1-mediated efflux, cholesterol can be effluxed from macrophages to HDL in an SR-BI-dependent pathway [96]. Conversely, the recovery of macrophage-derived label in serum and feces was not affected when mice were injected with macrophages lacking SR-BI [31,33]. Additionally, combined deletion of ABCA1 and SR-BI in macrophages did not impair macrophage RCT more than a single deletion of ABCA1 [33]. On the other hand, the effects of SR-BI overexpression in macrophages on RCT have not been investigated. In addition, bone marrow transplantation experiments consistently indicated a protective effect of SR-BI expression in macrophages on atherosclerotic plaque development [97-99]. Thus, the relevance of macrophage SR-BI for RCT is still debatable.

Another important player in cholesterol efflux and macrophage-specific RCT is apoE produced by macrophages. Cholesterol efflux from macrophages not expressing apoE was facilitated by endogenous expression of human apoE [100,101], whereas macrophages isolated from apoE knockout mice showed decreased cholesterol efflux elicited by HDL or lipid-free apoA-I *in vitro *[102]. In agreement, a recent study revealed that *in vivo *macrophage-to-feces RCT is diminished in wild-type mice receiving macrophages that are deficient in apoE [24].

As a final point, factors that regulate inflammatory responses in the macrophage may also be able to modify transport of cholesterol from the macrophage to the feces. Studies with a murine macrophage cell line transfected with the human 15(*S*)-lipoxygenase-1 gene demonstrated that human 15(*S*)-lipoxygenase-1 activity in macrophages accelerates cellular CE hydrolysis and consequently cholesterol efflux, leading to a net increase in macrophage RCT [37]. More surprisingly, the macrophage myeloid differentiation primary response protein 88, which is an adaptor protein involved in signal transduction of all toll-like receptors (TLRs) except TLR 3 and 4, exerts a positive effect on the RCT pathway at least partly through the upregulation of ABCA1 expression [38].

### Which proteins impacting cholesterol transport through the plasma compartment are relevant for reverse cholesterol transport?

A second essential determinant of efficient cholesterol elimination from macrophage foam cells is the amount of acceptors, principally apoA-I and HDL, present in the circulation. Overexpression of human apoA-I in mice resulted in more cholesterol being removed from macrophages and deposited in the feces via the RCT pathway lending strong support to the concept that raising HDL levels protects against atherosclerotic CVD at least in part by increasing RCT [18]. A subsequent study confirmed the specific contribution of apoA-I, independent of HDL, to macrophage RCT. When apoA-I was knocked out in the atherosclerosis prone LDLr^-/-^/apobec^-/-^mouse model, *in vivo *RCT was delayed [39]. Furthermore, macrophage-specific RCT can be improved in apoA-I-deficient mice by liver-directed expression of mouse or human apoA-I [40,41]. Consistent with these results, enhancement of apoA-I production in the liver of human apoA-I transgenic mice by treatment with the thienotriazolodiazepine Ro 11-1464 was accompanied by a greater flux of radiolabeled cholesterol from macrophages to stool [42]. Besides apoA-I plasma concentrations, also the tertiary structure domain of the protein appears to be an important determinant of its ability to promote RCT from macrophages [40]. On the other hand, the natural occurring apoA-I mutant apoA-I Milano, thought to exhibit superior atheroprotective effects, was equally effective in stimulating macrophage RCT than wild-type apoA-I [41]. Further proof for the capacity of apoA-I to facilitate RCT came from research using pharmacological agents mimicking apoA-I. Administration of the apoA-I mimetic peptides D-4 F [43], 5A [17], or ATI-5261 [44] to mice all increased the transfer of macrophage-derived cholesterol to plasma and feces. However, currently no data on the impact of infusing reconstituted HDL on RCT are available, although this intervention represents a promising clinical approach in patients [103,104].

The association between HDL cholesterol levels and macrophage-specific RCT is less straightforward. Hepatic [105] and intestinal [106] ABCA1 are crucial for HDL particle maturation, and mice with targeted deletion of ABCA1 have almost no circulatory pool of HDL [107]. In agreement with the lack of HDL, ABCA1 knockout mice exhibit an overall defect in macrophage-specific RCT [33,45]. However, the anti-atherosclerotic compound probucol, that inhibits hepatic ABCA1 activity and thereby reduces HDL cholesterol, had no effect on macrophage RCT in wild-type mice and even increased the flux through the macrophage RCT pathway on the SR-BI knockout background [46]. As one possible explanation the authors hypothesized that treatment with probucol targeted HDL-derived cholesterol taken up into the liver for biliary excretion by preventing hepatic ABCA1-mediated resecretion of cholesterol into the circulation [46]. Another example of a dissociation between plasma HDL cholesterol levels and macrophage RCT are SR-BI knockout mice [57]. Thus, plasma HDL levels do not necessarily represent a reliable reflection of macrophage RCT rates, and for that reason HDL cholesterol levels should be used with caution as a surrogate for predicting fluxes through the RCT pathway.

Preservation of a free cholesterol concentration gradient between the cell membrane and HDL due to esterification of cholesterol in HDL by LCAT is believed to support cholesterol efflux [12]. Paradoxically, LCAT activity does not seem to determine overall macrophage-to-feces RCT. In human apoA-I transgenic mice enhanced LCAT activity raised HDL levels, but failed to increase macrophage RCT [47]. In addition, heterozygous LCAT knockout mice also do not show a phenotype regarding macrophage-specific RCT [47]. On the other hand, LCAT deficiency in mice was associated with very low concentrations of HDL in the circulation, whereas the transfer of cholesterol from macrophages to feces *in vivo *was only decreased by approximately 50% in comparison to controls [47]. Moreover, in a recent report there was no correlation at all between the LCAT cholesterol esterification rates and the amount of macrophage-derived labeled cholesterol recovered in the feces [40].

Hepatic lipase (HL) and endothelial lipase (EL) are both negative regulators of HDL metabolism [108]. HL and EL knockout mice as well as HL/EL double knockouts have higher HDL cholesterol levels than their wild-type counterparts but decreased uptake of HDL-derived cholesterol into the liver. As a consequence the transport of cholesterol from macrophages to feces remains unchanged [48]. Nonetheless, an indirect increase in EL activity in mice via inhibition of hepatic proprotein convertases reduced HDL levels and resulted in a decreased disposal of macrophage-derived cholesterol into the feces [49]. In the case of the two lipases HL and EL, not the plasma HDL levels but the uptake of cholesterol from HDL into the liver appears to be rate-limiting for the macrophage RCT pathway.

Phospholipid transfer protein (PLTP) is another important enzyme involved in the remodeling of HDL. PLTP activity generates large HDL particles resulting in the release of poorly lipidated apoA-I [109]. Mice with transgenic overexpression of human PLTP display lower HDL concentrations and a decreased mobilization of radiolabeled cholesterol from peritoneal macrophages [34], suggesting that systemic PLTP activity impairs RCT.

CETP is highly relevant for human lipoprotein metabolism. Since inhibition of CETP raises circulating levels of HDL, which hypothetically should decrease the CVD risk amongst others by stimulating RCT, inhibition of CETP has been put forward as a novel therapeutic strategy. By facilitating the transfer of CEs from HDL to apoB-containing lipoproteins, CETP directs hepatic uptake of cholesterol to the LDL receptor, which might then represent an important route in the RCT pathway. Available research regarding the consequences of CETP activity for the atheroprotective RCT pathway has provided ambiguous data, as both elevation as well as inhibition of CETP activity can be favorable. Systemic expression of CETP by a recombinant adenoviral vector in wild-type mice [35] as well as long-term AAV-mediated expression of human CETP in apobec-1 knockout mice [50] led to a greater net transfer of radiolabeled cholesterol from macrophages to feces, and this favorable CETP modulatory effect on RCT required the presence of the LDL receptor [50]. In contrast, other studies found no evidence that CETP influences macrophage-to-feces RCT [36,51]. In hamsters, which naturally express CETP, treatment with the potent CETP inhibitor torceptrapib or anacetrapib to some extent improved the movement of cholesterol from macrophages in the peritoneal cavity to the feces [35,52,54], although with anacetrapib this was only observed under dyslipidemic conditions [52,54]. In addition, in human CETP/human apoB100 transgenic mice on a high-fat diet administration of torcetrapib increased HDL-C levels and enhanced RCT from macrophages to feces [53]. Besides CETP inhibition, specific modulation of CETP activity by dalcetrapib in hamsters was also associated with a higher magnitude of macrophage RCT [52]. Overall, the position of CETP in RCT appears to be complex and requires in our view further accurate investigation, especially in light of the growing interest in the clinical use of CETP inhibitors.

Macrophage RCT may also be impacted by the specific apolipoproteins carried in the HDL particle. ApoA-II, for example, is the second major apolipoprotein in HDL [110]. Expression of human apoA-II did not impair macrophage-specific RCT in mice fed either a chow or an atherogenic diet, despite a pronounced lowering of plasma HDL-C levels in response to human apoA-II expression on both diets [55]. An elevated content of apoF in HDL, in terms of mass a minor constituent of the particle, enhanced its capacity to serve as an acceptor for macrophage cholesterol, but this did not translate into higher macrophage-specific RCT *in vivo *[56].

### What is the importance of cholesterol uptake by the liver for reverse cholesterol transport?

Following transport through the plasma compartment, the next step in RCT is delivery of cholesterol from macrophages to the liver. SR-BI is the key receptor responsible for the selective uptake of CEs from HDL into the liver, and hepatic SR-BI has been recognized as a positive regulator of RCT [57]. Consistent with the effects on experimental atherosclerosis, hepatic SR-BI overexpression resulted in more macrophage-derived cholesterol being excreted into the feces [57], whereas macrophage RCT is clearly impaired in the total absence of SR-BI [33,57] as well as when SR-BI is exclusively deleted in the liver [51]. Although one study suggested that introduction of CETP can correct the adverse phenotype regarding macrophage RCT in SR-BI knockout mice by shuttling HDL-associated CEs to apoB-containing lipoproteins for receptor-mediated hepatic uptake [50], this was not confirmed by subsequent research [51]. These differences might be related to the means of CETP overexpression used in these studies, either by AAV [50] or by transgenic overexpression using a construct with the natural flanking regions [51].

An alternative mechanism by which HDL cholesterol can be taken up into the liver is via holoparticle endocytosis, i.e. uptake of both HDL proteins and lipids at an equal rate. Although the definitive receptor mediating this has not been identified thus far, it was noted that the P2Y_13 _receptor is involved in HDL holoparticle uptake [111]. Mice that lack the P2Y_13 _receptor exhibit a substantial reduction in HDL holoparticle uptake into the liver, and as a result the fecal excretion of cholesterol originating from macrophages is reduced [58].

However, enhanced uptake of HDL-derived cholesterol in the liver apparently only results in accelerated RCT when associated with increased biliary cholesterol secretion as it is the case for SR-BI and P2Y_13_[58,112,113]. Conversely, increased hepatic uptake of HDL cholesterol does not necessarily translate into changes in biliary secretion when the hepatic expression levels of SR-BI remain unaltered. For instance, mice overexpressing EL [112] or human group IIA secretory phospholipase A_2_[69,114] were noted to have elevated selective uptake of HDL CEs into the liver, but there was no concomitant increase in cholesterol removal via the bile or RCT. On the other hand, impaired hepatic selective uptake by modifying the donor properties of the HDL particle results in decreased RCT as we have recently shown in the case of insulin-deficient type 1 diabetic mice [59]. In this model, HDL glycation decreased SR-BI-mediated selective uptake translating into lower RCT rates despite enhanced biliary cholesterol mass secretion [59]. In addition, decreased RCT in type 1 diabetes was shown to be modified by the haptoglobin genotype with the haptoglobin 2-2 genotype resulting in an aggravated reduction [60].

### What is the importance of biliary versus non-biliary pathways for macrophage-derived cholesterol to enter the intestinal lumen?

Before HDL-derived CEs can be excreted into the bile, they first need to be hydrolyzed to generate free cholesterol. Similar to the macrophage, hepatic CE hydrolysis can be achieved by the action of nCEH. Adenoviral hepatic overexpression of nCEH increased RCT from macrophages to feces, primarily by augmenting the biliary output of bile acids [61]. Yet, mice with genetic deficiency of carboxyl ester lipase, which likewise has the capacity to hydrolyze CEs in the liver, unexpectedly show augmented secretion of HDL-CE as well as macrophage-derived cholesterol into bile and feces [62]. A satisfactory explanation for this discrepancy is currently not available.

Biliary secretion has classically been regarded the major route for elimination of RCT-relevant cholesterol from the body, although for a long time this concept had not been experimentally tested. Hepatic cholesterol can be secreted into bile either directly as free cholesterol or after conversion into bile acids. Biliary phospholipid secretion through the multi-drug resistance P-glycoprotein 2 (MDR2 or ABCB4) is obligatory for functional hepatobiliary cholesterol secretion, as phospholipid-induced formation of mixed micelles is key in the solubilization of cholesterol in bile (for a recent comprehensive review on the mechanisms of biliary cholesterol excretion please see [115]). Bile acids are secreted by the bile salt export pump (or ABCB11). ABCG5 and ABCG8 are obligate heterodimers that mediate secretion of cholesterol and plant sterols into bile together with the cholesterol-binding protein Niemann-Pick C2 (NPC2) [116]. Although the absence of ABCG5/G8 results in a marked reduction in biliary cholesterol secretion [117], RCT from macrophages was found to be unaltered in ABCG5/ABCG8 double knockout mice [63], while the role of NPC2 in RCT has not been explored, yet.

Since it had been noted that non-biliary pathways contribute to total fecal neutral sterol excretion [118-121], we experimentally tested the relevance of biliary sterol secretion for RCT. Following bile duct ligation RCT was almost completely abolished [64]. In addition to this surgical model, also in a non-cholestatic genetic model of virtually absent biliary cholesterol secretion, namely MDR2-deficient mice, there was a drastic reduction in RCT in fecal neutral sterols [64]. Interestingly, RCT via bile acids did not compensate for the severe reduction in RCT via neutral sterols in MDR2 knockout mice, and also the stimulating effects of LXR ligands on RCT depended largely on functional biliary cholesterol secretion [64]. Of note, we observed a clear distinction between fecal neutral sterol mass changes and macrophage-derived tracer counts suggesting different metabolic pathways. Furthermore, the results of HDL kinetic studies conducted in parallel to the RCT experiments were counterintuitive to the intestine playing a major role in RCT [64]. These combined results led us to the conclusions that, at least in the models tested, the biliary secretion pathway was of primary importance for functional *in vivo *RCT. However, using a different experimental approach Temel *et al. *showed that transgenic mice expressing human Niemann-Pick C1-like 1 (NCP1L1) in the liver have substantially reduced cholesterol concentrations in gallbladder bile but exhibit no apparent deficit in macrophage-specific RCT [65]. In addition, in a very short-term experiment RCT did not differ significantly between bile duct diverted mice and controls [65]. Although in contrast to bile duct ligation bile duct diversion has the advantage of not inducing cholestasis, bile acids, however, also do not enter the intestinal lumen. Since in the initial studies on this pathway bile acids have been shown to be essential as acceptors for intestinal cholesterol excretion, the nature of the cholesterol acceptors in the bile duct diversion experiments remains unclear. Differences between the two studies other than the models used also comprise the choice of macrophages, primary mouse peritoneal macrophages [64] versus the J774 cell line [65]. However, in summary, the contrasting results obtained can as yet not be explained. Therefore, a definitive answer to the question about a contribution of the intestine to RCT has to await (i) the clarification that the intestinal cholesterol excretion pathway is indeed an active metabolic process, (ii) the delineation of the molecular identity of the intestinal transporters involved, and (iii) the characterization of the lipoprotein substrates relevant for this pathway.

### What is the impact of intestinal absorption on reverse cholesterol transport?

The transport protein NPC1L1 is highly relevant for the intestinal uptake of cholesterol [122] and has been identified as the molecular target of the cholesterol absorption inhibitor ezetimibe [123]. Inhibition of intestinal cholesterol absorption using ezetimibe in mice resulted in increased RCT [66,67]. Furthermore, experiments in a congenic mouse strain with genetically lowered cholesterol absorption revealed that even a moderate decrease in the amount of cholesterol absorbed from the intestinal lumen is associated with increased RCT [67,68]. Opposite to NPC1L1, the half-transporters ABCG5 and ABCG8 may participate in the active transport of cholesterol from the enterocyte back into the intestinal lumen permitting fecal excretion [124]. However, to date the specific involvement of intestinal ABCG5/ABCG8 in RCT has not been explored.

## What are the factors influencing multiple steps in the reverse cholesterol transport pathway?

In addition to factors that predominantly affect one single step, there are also factors and compounds that influence multiple steps in the macrophage-specific RCT pathway such as (i) inflammation, (ii) various drugs, (iii) dietary modifications, and (iv) exercise (please see also Table 1 for a summary).

### What is the impact of inflammation on reverse cholesterol transport?

Inflammation plays a central role in atherogenesis, and there is good evidence that inflammation decreases RCT. Acute inflammation induced by a single lipopolysaccharide (LPS) injection profoundly hampered the movement of labeled cholesterol from macrophages to the plasma and feces in wild-type mice [69,70]. In addition, diminished *in vivo *RCT has also been detected after an inflammatory response elicited by the yeast cell wall extract zymosan [71], although this effect was substantially lower than the impact of LPS on RCT. What are the steps in RCT affected by an inflammatory response? A reduced efflux capacity of acute-phase HDL might be involved, as evidenced in experimental murine and human endotoxemia [69,70] as well as in acute sepsis patients [69]. Furthermore, severely elevated plasma concentrations of the acute-phase proteins myeloperoxidase and serum amyloid A during inflammation have been identified as additional contributing factors [69]. Also the liver plays an important role, since during an acute phase response enzymes involved in the conversion of cholesterol to bile acids are down-regulated and the expression of transporters mediating biliary secretion of cholesterol and bile acids is severely decreased [69,70,125,126].

Mast cells in atherosclerotic lesions have been recognized to participate in the inflammatory processes that drive atherosclerotic plaque development [127]. A recent report suggested that degranulation of mast cells in the vascular wall may locally suppress cholesterol removal from macrophages, and activation of mast cells in the peritoneal cavity of mice completely abrogated the apoA-I-induced increase in RCT [72].

### Which effects do various drugs have on reverse cholesterol transport?

#### LXR agonists

LXRs are nuclear receptors activated by endogenous oxysterols that control genes involved in lipid metabolism and cholesterol transport, and therefore LXRs are in principal considered an attractive therapeutic target for atherosclerotic CVD [128]. A number of studies have examined the role of LXR in macrophage-to-feces RCT and consistently found a higher flux through this pathway following pharmacological LXR activation in CETP-deficient as well as CETP-expressing animals [63,73-76]. Several mechanisms apparently contribute to LXR-mediated activation of RCT. Firstly, LXR upregulates the expression of ABCA1 and ABCG1 in macrophages and has been shown to stimulate macrophage cholesterol efflux *in vitro *[129,130]. Macrophage LXR is important in the ability of LXR to promote RCT, but is not vital. Although in LXR agonist-treated wild-type mice injected with macrophages from LXR double knockout mice RCT was lower compared with similar treated wild-type mice injected with wild-type macrophages, LXR activation still promoted RCT in the absence of macrophage LXR [73]. On the other hand, activation of LXR restricted to macrophages was inadequate to increase RCT [73]. Secondly, LXR may improve the potential of plasma to accept cholesterol from macrophage foam cells by increasing plasma HDL cholesterol levels [75]. Thirdly, pharmacological LXR activation induces expression of *Abcg5 *and *Abcg8 *in the liver [63,73,74,76], most likely resulting in an increased elimination of cholesterol via the biliary route. Modulation of macrophage RCT by a synthetic LXR ligand required functional biliary cholesterol secretion, as its effect was abolished in ABCG5/ABCG8 double knockout [63] as well as MDR2 knockout mice [64]. However, unaltered macrophage-to-feces RCT in response to adenovirus-mediated hepatic overexpression of LXRα in mice supported a less important role of the liver in LXR-mediated effects on RCT [77]. Fourthly, LXR activation in the small intestine inhibits cholesterol absorption [77] via induction of *Abcg5 *and *Abcg8 *as well as downregulation of *Npc1l1 *expression [73,74,76,77]. Consistent with these data, RCT was increased in transgenic mice specifically overexpressing LXR in the intestine [77] and after treatment of mice with an intestinal-specific LXR agonist [74].

#### PPAR agonists

Peroxisome proliferator-activated receptors (PPARs) are transcription factors that like LXRs belong to the nuclear receptor family and modulate expression of genes implicated in several biological processes such as lipid metabolism, glucose metabolism, and inflammation [131,132]. Three members of the PPAR family have been identified (PPARα, PPARδ, and PPARγ), which have a distinct tissue distribution and modulate different biological responses after activation (for detailed reviews please see [133-135]). Lately, PPAR ligands have attracted interest in view of their potential use for treatment of cardiovascular diseases. Both in humans [136-138] and experimental animals [139-142] activation of PPARs has been associated with a raise in plasma HDL cholesterol levels, which in theory might improve RCT.

Indeed, recent studies revealed that the potent PPARα agonist GW7647 increased macrophage RCT in a hyperlipidemic mouse model expressing human apoA-I [78]. Analysis of the molecular mechanism revealed that GW7647 stimulated cellular cholesterol efflux and correspondingly the RCT pathway by up-regulation of ABCA1 and ABCG1 in macrophages via a PPARα-LXR-dependent pathway [78]. A similar advantageous outcome on overall RCT was observed in human apoA-I transgenic mice receiving the PPARα ligand fenofibrate, though this effect was restricted to female mice [79]. Moreover, off-target effects on RCT by fenofibrate cannot be excluded, given that in the same animal model another fibrate, gemfibrozil, equally increased PPARα activation in the liver without a concomitant enhancement in RCT [79].

Dietary supplementation with a PPARδ-specific agonist was associated with an elevated level of macrophage-derived tracer excreted into feces of wild-type mice [66]. Compared with PPARα, which has been shown to modify *in vivo *RCT at the macrophage level [78], PPARδ-mediated effects on the macrophage RCT pathway seem largely confined to the intestine [66]. PPARδ activation in mice led to a decreased intestinal expression of *Npc1l1 *[66,141] and as a consequence diminished the capacity of the intestine to absorb cholesterol [141].

Finally, PPARγ agonists were developed for therapeutical use in type 2 diabetes mellitus. Interestingly, treatment of wild-type mice with a synthetic PPARγ agonist considerably impeded RCT from macrophages to feces [80]. Using kinetic experiments, the authors showed that PPARγ activation promoted SR-BI-mediated uptake of cholesterol from HDL into the adipose tissue [80], shunting cholesterol away from the liver and thus likely reducing biliary elimination although this was not experimentally addressed.

### Other drugs and therapeutic modalities

Likewise a number of other drugs have been tested with the macrophage-specific RCT method. The farnesoid X receptor (FXR) has been implicated in the control of cholesterol metabolism through transcriptional regulation of several genes, including *ApoA-I*, *Cyp7a1*, *Pltp*, *ApoC-II*, and *ApoC-III *[143]. Activation of FXR, by treatment with the specific agonist GW4064 or an adenovirus expressing constitutively active FXR, enhanced transport of cholesterol from macrophages to feces in wild-type mice in the face of lower HDL levels [81]. This was partially SR-BI-dependent, since the effects of the FXR agonist on RCT were attenuated in SR-BI knockout mice [81]. However, the dependency of these results on functional FXR expression have not formally been addressed.

Recently, the microRNA miR-33, that is expressed from an intron within the SREBP-2 gene, has been identified as an important repressor of the cholesterol transport genes *Abca1 *and *Abcg1 *[144,145]. Inhibition of miR-33 in LDL receptor knockout mice by antisense oligonucleotides raised circulating HDL and promoted the macrophage RCT pathway, which in turn may have contributed to the regression of pre-established atherosclerosis observed in anti-miR33-treated mice [82].

Another potential anti-atherogenic drug, the liver-selective thyromimetic T-0681, reduced plasma levels of cholesterol and stimulated delivery of macrophage-derived cholesterol into the feces in wild-type mice [83]. These findings are consistent with the atheroprotective effect of T-0681 in apoE knockout mice upon prolonged treatment [83]. Nevertheless, it is unclear if similar results are to be expected in humans, as macrophage RCT remained unchanged in T-0681-treated CETP transgenic mice [83].

Glibenclamide and glimepiride are sulfonylurea agents widely used to treat insulin resistance, and administration of either one of these drugs to wild-type mice did not alter RCT [84].

Finally, the anti-HIV drugs efavirenz and nevirapine [85], the selective inhibitor of phosphodiesterase 3 cilostazol [86], the proteasome inhibitor bortezomib [87], and ethanolic extracts of propolis [88] have all been demonstrated to favorably influence macrophage-to-feces RCT.

### What is the impact of diet on reverse cholesterol transport?

Diets with increased fat and/or cholesterol contents are generally used in experimental animal models to induce atherosclerotic lesion development, and also in humans a high intake of dietary saturated fatty acids and cholesterol has been associated with an increased risk of mortality from coronary heart disease [146]. Increased plasma levels of atherogenic lipoproteins is most likely the major contributing factor to the initiation of plaque formation by dietary modification. Although published results are ambiguous, impaired RCT might also play a role. When hamsters with endogenous CETP expression were fed a diet containing 0.3% cholesterol for 4 weeks to induce dyslipidemia, a pronounced reduction in overall RCT was observed [89]. Concomitantly, the cholesterol-rich diet impaired the capacity of plasma to promote release of cholesterol from macrophages, consistent with the decrease in macrophage RCT [89]. In contrast, studies in wild-type mice and human CETP-transgenic mice suggested a stimulating effect of a diet high in both saturated fatty acids and cholesterol on macrophage cholesterol efflux to plasma as well as *in vivo *RCT [90]. The increased RCT in response to a high fat/high cholesterol diet in mice was apparently dependent on dietary cholesterol and functional expression of *Abcg5/g8 *[90].

Also individual dietary components may impact RCT. Ferulic acid is an abundant polyphenol in coffee with antioxidant properties, and treatment of wild-type mice with ferulic acid increased macrophage-specific RCT by inducing the expression of ABCG1 and SR-BI in macrophages, thereby promoting HDL-mediated cholesterol efflux [91]. However, coffee intake itself did not lead to a change in macrophage RCT in mice [91].

A diet enriched in fish oil has been shown to enhance macrophage RCT in mice as compared to diets rich in other sources of fatty acids [92]. Increased excretion of HDL-derived cholesterol from the body, attributable to decreased esterification of cholesterol in the liver, increased hepatic expression of *Abcg5 *and *Abcg8*, and decreased intestinal expression of *Npc1l1*, was suggested to account for this elevated rate of RCT by dietary fish oil [92].

### Does physical exercise impact reverse cholesterol transport?

Physical exercise is suggested as a preventive strategy against CVD, and exercise increases fecal mass excretion of neutral sterols and bile acids [93,147]. However, a study by our group did not find any impact of voluntary wheel running on *in vivo *macrophage RCT in wild-type mice, even though cholesterol efflux from macrophage foam cells towards plasma of exercising mice *in vitro *was significantly increased [93]. On the other hand, macrophage RCT was higher in exercising human CETP transgenic mice when compared with sedentary controls [94]. This beneficial effect of regular exercise training on RCT was at least in part ascribed to a raise in plasma HDL cholesterol and an enhanced hepatic uptake of cholesterol through elevated LDL receptor protein expression [94]. The difference between these two studies might be due to either the exercise protocol (voluntary [93] versus forced [94]), the use of macrophages (primary [93] versus cell line [94]) or the expression of CETP, but unfortunately the latter study lacked wild-type controls not expressing CETP.

## Concluding remarks and future directions

• RCT represents a relevant atheroprotective pathway that is, however, only one piece in a complex mechanistic network determining atherosclerotic lesion formation, progression and regression. To date, formal causal evidence is lacking that RCT quantified by the methods described in this review reflects the actual dynamics of the process of atherogenesis.

• Despite a vast amount of experimental data gathered to date, it remains unclear whether cholesterol movement through the entire RCT pathway is required for atheroprotection. Mobilization of cholesterol from macrophages might be sufficient in this respect, at least in an acute clinical setting. However, since effluxed cholesterol can be redistributed to the vessel wall from other tissues, in our opinion increasing the fecal sterol excretion of macrophage-derived cholesterol together with lowering of apoB-containing lipoproteins constitutes the favorable strategy.

• In our view, valuable pathway information can be derived from macrophage RCT studies by distinguishing within the feces between counts in the neutral sterol versus the bile acid fractions, which is thus far not consistently done.

• We would also like to stimulate putting RCT studies in a broader metabolic context by combining these with mass measurements of sterol excretion.

• Finally, a reliable method for quantifying macrophage RCT in humans would be a valuable tool for clinical drug development and translational studies.

## Abbreviations

ABC: ATP binding cassette transporter; Apo: Apolipoprotein; CE: Cholesteryl ester; CETP: Cholesteryl ester transfer protein; CVD: Cardiovascular disease; EL: Endothelial lipase; FXR: Farnesoid X receptor; HDL: High density lipoproteins; HDL-C: High density lipoprotein cholesterol; HL: Hepatic lipase; LCAT: Lecithin-cholesterol acyltransferase; LDL: Low density lipoproteins; LPS: Lipopolysaccharide; LXR: Liver X receptor; MDR2: Multi-drug resistance P-glycoprotein 2; nCEH: Neutral cholesteryl ester hydrolase; NPC1L1: Niemann-Pick C1-like 1; NPC2: Niemann-Pick C2; PLTP: Phospholipid transfer protein; PPAR: Peroxisome proliferator-activated receptor; RCT: Reverse cholesterol transport; SR-BI: Scavenger receptor class B type 1; TLR: Toll-like receptor.

## Competing interests

The authors declare that they have no competing interests.

## Authors' contributions

WA and UJFT reviewed the literature and drafted the manuscript. Both authors read and approved the final manuscript.
